# Effectiveness of health exhibitions on knowledge and attitudes toward nutrition and disease prevention in Saudi Arabia: an intervention study

**DOI:** 10.3389/fpubh.2026.1827711

**Published:** 2026-08-07

**Authors:** Yara Alromaih, Sundus Aldakhil, Deem Alsaloom, Ghadi Abdullah Alkhalaf, Roba Alshehi

**Affiliations:** Research and Studies Unit, Patients Friends Association in Unaizah, Unaizah, Al-Qassim, Saudi Arabia

**Keywords:** community intervention, health exhibitions, nutrition knowledge, public health awareness, Saudi Arabia, vaccine education

## Abstract

Although health exhibitions are interactive platforms for large audiences, few studies have evaluated their effectiveness in improving participants’ knowledge in Saudi Arabia. In this study, we aimed to evaluate the effect of health exhibitions on public health knowledge and attitudes among adult residents of Unayzah, Saudi Arabia, focusing on the “Vaccine Preventable Disease Awareness Exhibition” and the “Health and Nutrition Exhibition,” which took place from January to April 2025. This study employed a one-group pretest–posttest quasi-experimental design. Data were collected using a self-administered electronic questionnaire from 389 participants at the Vaccine Preventable Disease Awareness Exhibition and 162 participants at the Health and Nutrition Exhibition. We assessed changes in participants’ knowledge and attitudes toward nutrition, disease prevention, and vaccination by comparing pre- and post-exhibition assessments. We explored the influence of demographic factors, such as age, sex, and education level, on participants’ engagement and knowledge acquisition. As data were non-normally distributed, non-parametric tests were applied: McNemar’s test for paired categorical knowledge items, Wilcoxon signed-rank test for attitude scores, and Kruskal–Wallis test with Dunn–Bonferroni post-hoc comparisons for demographic analyses. Participants’ knowledge of health and nutrition and their perceptions of vaccines improved after the exhibitions. There were significant differences between pre- and post-assessment knowledge of the E100 food additive symbol (*p* < 0.001), meal-skipping effects (*p* < 0.001), and gout-related dietary knowledge (*p* < 0.001), as well as vaccination knowledge items including cause of plague (*p* < 0.001) and vaccine-eradicated diseases (*p* < 0.001). Our findings revealed that both exhibitions enhanced knowledge in the target community, underscoring the role of community health education in improving public awareness. This study provides insight into the short-term effect of interactive exhibitions on public health knowledge and attitudes.

## Highlights

- This study provides insight into the short-term effect of health exhibitions on public knowledge and attitudes toward nutrition and disease prevention.
- The exhibitions enhanced knowledge in the target community, indicating that community health education improves public health awareness and knowledge.
- The exhibitions proved effective in strengthening public understanding of infectious disease transmission and prevention, highlighting the role of interactive community-based education in correcting misconceptions and reinforcing evidence-based health knowledge.

## 1 Background

The modern public health system is a multidisciplinary field that aims to improve health and reduce health inequalities at the organizational, community, and societal levels (1). Community education is a fundamental strategy for influencing lifestyle behaviors (2). Nowadays, different public education strategies, such as health campaigns, mass media programs, seminars and talks, workshops, and school-based health education programs, can be implemented to improve community health. Additionally, health fairs, carnivals, and exhibitions are health promotion strategies that help healthcare providers reach large numbers of people in their communities. Information collected from health exhibitions can provide insight into community demographics, health outcomes, and social determinants of health (3, 4). This information can improve community-based initiatives by determining the appropriate areas for resource allocation. However, organizing content and developing programs or services that reach and resonate with the intended audience and evaluating the impact of these programs are a challenge in community health education (2). A critical aspect of health promotion efforts is the evaluation of their impact, not only in terms of the outcomes achieved but also the processes through which those outcomes are attained, especially in complex, community-based settings (5). Although various initiatives have been launched in Saudi Arabia, comprehensive assessments are lacking, making it difficult to determine their overall effectiveness and utilization rates among different demographic segments. Evaluating these programs not only assists in identifying best practices but also supports the optimization of future health interventions (6).

Infectious diseases remain a major public health challenge, with vaccine hesitancy emerging as a significant issue in Saudi Arabia, particularly during the COVID-19 pandemic (7, 8). Social media has played a pivotal role in spreading false information about vaccinations (9, 10), and studies suggest that vaccine hesitancy often stems from a lack of understanding of vaccine safety profiles (8). Despite extensive efforts by the Saudi Ministry of Health, including national campaigns promoting influenza vaccination, research indicates a rise in vaccine reluctance post-pandemic (7, 8). Health education campaigns emphasizing the benefits of vaccines have improved public acceptance, making them a critical tool in addressing this issue. Antibiotic use is another critical issue in the medical field. Despite progress in the development of effective antibiotics to treat various ailments, antibiotic resistance appears to be on the rise. Although stricter regulations have led to a decrease in non-prescription antibiotic use (11), misconceptions about antibiotics persist. Many individuals believe that antibiotics are effective against viral infections, and some discontinue its use prematurely, contributing to resistance (12). Awareness campaigns can help educate the public about the dangers of inappropriate antibiotic use and its broader implications for public health (13). By establishing educational programs and adhering to the World Health Organization (WHO) recommendations, communities must raise awareness of antimicrobial resistance and curb the growth of antimicrobial resistance in the Arab world. The WHO Global Action Plan for Influencing Behavior Change strongly emphasizes the importance of educating stakeholders and distributing information to increase awareness of antibiotic resistance (14).

Health awareness is the ability of individuals and communities to obtain and apply knowledge in ways that support good health. In Saudi Arabia, unhealthy eating habits, food misinformation, and inadequate nutritional knowledge remain growing public health concerns (15, 16). Health exhibitions, held in public spaces or educational institutions, provide an interactive platform for direct knowledge transfer between healthcare professionals and the community, and have demonstrated efficacy in improving public awareness on topics such as cancer prevention and general health promotion (3, 17).

In Saudi Arabia, despite the growing popularity of health exhibitions, limited research has been conducted on their effect on public knowledge and attitudes. To address this gap, we conducted two exhibitions—the “Vaccine Preventable Disease Awareness Exhibition” and the “Health and Nutrition Exhibition”—held in Unayzah from January to April 2025, with the aim of evaluating their immediate effect on attendees’ knowledge and attitudes by comparing pre- and post-exhibition assessments, and providing evidence-based recommendations for improving public health awareness through interactive community platforms.

## 2 Methods

### 2.1 Ethical considerations

This study was approved by the local research committee in the Qassim region (approval number: 607/46/7797 and date: 3/1/2025). Informed consent was obtained from each participant prior to their enrollment in the study. Participants’ data were kept confidential and used only for research purposes. The study was carried out following the principles of the Declaration of Helsinki for research with human volunteers.

### 2.2 Study design

*Study type*: We adopted a one-group pretest–posttest quasi-experimental design, utilizing pre-and post-test assessments to evaluate the immediate effect on attendees’ knowledge and attitudes regarding Vaccine Preventable Disease and nutrition topics. This design is appropriate for community-based educational interventions where randomization or a control group is not feasible, as it allows for the evaluation of within-group changes following exposure to a structured health intervention.

*Sample size*: The study did not have a predetermined sample size. Instead, it included all participants who attended at least one of the exhibitions: the “Vaccine Preventable Disease Awareness Exhibition” and the “Health and Nutrition Exhibition,” both held in Unayzah in January 2025. Participants were selected using a convenience sampling approach. To reduce selection bias trained health educator systematically invited every eligible visitor to the exhibitions to participate. Data collection was conducted over many days and at varying times to ensure a demographically diverse sample. Overall, we included 389 participants from those who fully completed both pre- and post-test questionnaires across both exhibitions.

*Study setting*: The “Vaccine Preventable Disease Awareness Exhibition” and the “Health and Nutrition Exhibition” were organized in Unayzah and took place from January to April 2025.

*Inclusion criteria*: No formal clinical screening was conducted to identify underlying chronic or neurological conditions due to the nature of the field setting. However, individuals who appeared to have visible signs of cognitive impairment or who were unable to engage meaningfully in the activity (as judged through observation by trained staff) were respectfully not included in the data collection process. This approach was taken to ensure reliability of the responses.

Questionnaires with major missing sections or incomplete answers were excluded from the final analysis. Only participants who completed both pre- and post-test questions were included in the statistical evaluation to preserve data integrity.

### 2.3 Study tools

A self-administered questionnaire was used to evaluate the knowledge of visitors before and after attending the exhibitions. Custom-designed questionnaires were developed specifically to assess the impact of the two exhibitions. The questionnaires were provided in Arabic language and the result were translated to English. A pre-exhibition test was administered before to determine their baseline knowledge and attitudes regarding health, nutrition, and vaccinations. After completing the exhibition a post-exhibition test was administered to assess changes in their knowledge and attitudes and compared with the pre-test.

Two questionnaires, each consisting of 12 questions, were used. Both questionnaires were divided into two sections. Knowledge scores were computed as the sum of correct responses, ranging from 0 to 3 for the vaccination questionnaire and 0 to 4 for the nutrition questionnaire. Attitude scores were calculated as the sum of three-point Likert-scale responses (0 = disagree, 1 = neutral, 2 = agree) across all attitude items, ranging from 0 to 10 for the vaccination questionnaire and 0 to 12 for the nutrition questionnaire, with higher scores reflecting more favorable attitudes.

### 2.4 Demographic data

This section includes questions (1–4) on sex, age group, educational level, and the main sources of information and news.

### 2.5 Knowledge and attitude related questions

The first questionnaire focused on nutrition and health. Questions 5–9 were knowledge-related and questions 10–12 were attitude-related. Attitude-related questions were evaluated using the following responses: agree, neutral, and disagree. The second questionnaire addressed Vaccine Preventable Diseases. Questions 5–7 were knowledge-related, while questions 8–12 were attitude-related questions.

### 2.6 Variables

The dependent variables were health knowledge regarding infectious diseases and prevention (Vaccine Preventable Disease Awareness Exhibition), nutritional knowledge of healthy eating and associated health risks (Health and Nutrition Exhibition), and attitudes toward vaccinations and healthy eating after exposure to the exhibitions. The independent variables were age, sex, and educational level.

Although the main sources of information and news were collected as a demographic variable and reported descriptively, this variable was not included as an independent variable in the inferential analyses. This decision was based on the multi-select nature of the question, which precluded straightforward group-based comparisons, and the fact that it was not a primary demographic characteristic of interest in the pre-specified research objectives.

### 2.7 Data collection procedures

Exhibition materials, such as interactive displays, were designed to encourage participant engagement through hands-on activities, ensuring the accommodation of diverse learning styles. At the entrance of each exhibition, participants were informed about the research objectives and invited to voluntarily participate in the study. Those who agreed to participate scanned a quick response code to access the electronic pre-test. The pre-test took approximately 5–10 min to complete.

During the exhibition, health educators guided the participants through various interactive displays, including videos, holograms, and 3D models. The exhibition experience lasted approximately 30–45 min, allowing sufficient time for participants to interact with all the materials.

Immediately after completing their exhibition tour, participants were prompted to fill out the post-test electronically. This post-test measured changes in their knowledge and attitudes, ensuring alignment with the pre-test for consistency and comparability. The post-test also took approximately 5–10 min to complete.

### 2.8 Statistical analysis

Data were analyzed using IBM SPSS Statistics (Version 25; IBM Corp., Armonk, NY, USA). For categorical variables, descriptive statistics were expressed as frequencies and percentages, and for continuous score variables where appropriate, we presented the means ± standard deviations (SDs). The normality of knowledge and attitude scores before and after the exhibition was checked using the Shapiro–Wilk test. The normality assumptions were not met for all score variables (*p* < 0.001). Levene test was used to check the homogeneity of variance. Variances were significantly heterogeneous for post-exhibition knowledge scores (*p* = 0.027) but not post-exhibition attitude scores (*p* = 0.059).

Data regarding outcome variables were ordinal or non-normal, and hence non-parametric statistical methods were utilized. McNemar’s test for paired categorical responses was used to assess changes in participants’ knowledge pre- vs. post-exhibition. Wilcoxon signed-rank test was performed to compare participants’ attitudes toward vaccination before and after the exhibition. The associations between demographic characteristics and post-exhibition knowledge and attitude scores were examined using the Kruskal–Wallis test. To adjust for multiple testing, Dunn–Bonferroni *post hoc* pairwise comparisons were performed following Kruskal–Wallis analyses where appropriate. A two-sided alpha of 0.05 was used to determine statistical significance. All reported *p*-values were two-tailed.

## 3 Results

A total of 389 participants completed the Vaccination questionnaire. The study included the following demographic information; 69.7% (*n* = 271) of the respondents were female and 30.3% (*n* = 118) were male. The largest age group was 21–30 years (43.4%, *n* = 169), followed by the 10–20 years age group (28.0%, *n* = 109). Almost 1 in 2 participants were university graduates (45.5%, *n* = 177). Vaccination-related information was inquired from the most reported sources, such as social media (21.9%, *n* = 85) and healthcare professionals (21.1%, *n* = 82; Tables 1, 2).

**Table 1:** Demographic characteristics of participants with vaccination-based questionnaire.

Variable	Category	*n*	%
Sex	Female	271	69.7
	Male	118	30.3
Age group (years)	10–20	109	28.0
	21–30	169	43.4
	31–40	60	15.4
	41–50	32	8.2
	>50	19	4.9
Education level	Primary	10	2.6
	Middle school	31	8.0
	High school	100	25.7
	Diploma	51	13.1
	Bachelor’s degree	177	45.5
	Master’s degree	12	3.1
	PhD	6	1.5
	Uneducated	2	0.5

**Table 2:** Normality and homogeneity testing of knowledge and attitude scores.

Variable	Shapiro–Wilk *W*	*p*-value
Knowledge score (pre)	0.919	<0.001
Knowledge score (post)	0.752	<0.001
Attitude score (pre)	0.848	<0.001
Attitude score (post)	0.708	<0.001

The Shapiro–Wilk test was used to test for normality of knowledge and attitude scores pre and post- exhibition. All variables were significantly deviated from a normal distribution (all *p* < 0.001). Homogeneity of Variance was valuated using Levene’s test. Levene’s test indicated unequal variances for post-exhibition knowledge scores (*p* = 0.027), whereas post-exhibition attitude scores did not significantly violate the homogeneity assumption (*p* = 0.059).

**Figure 1:**
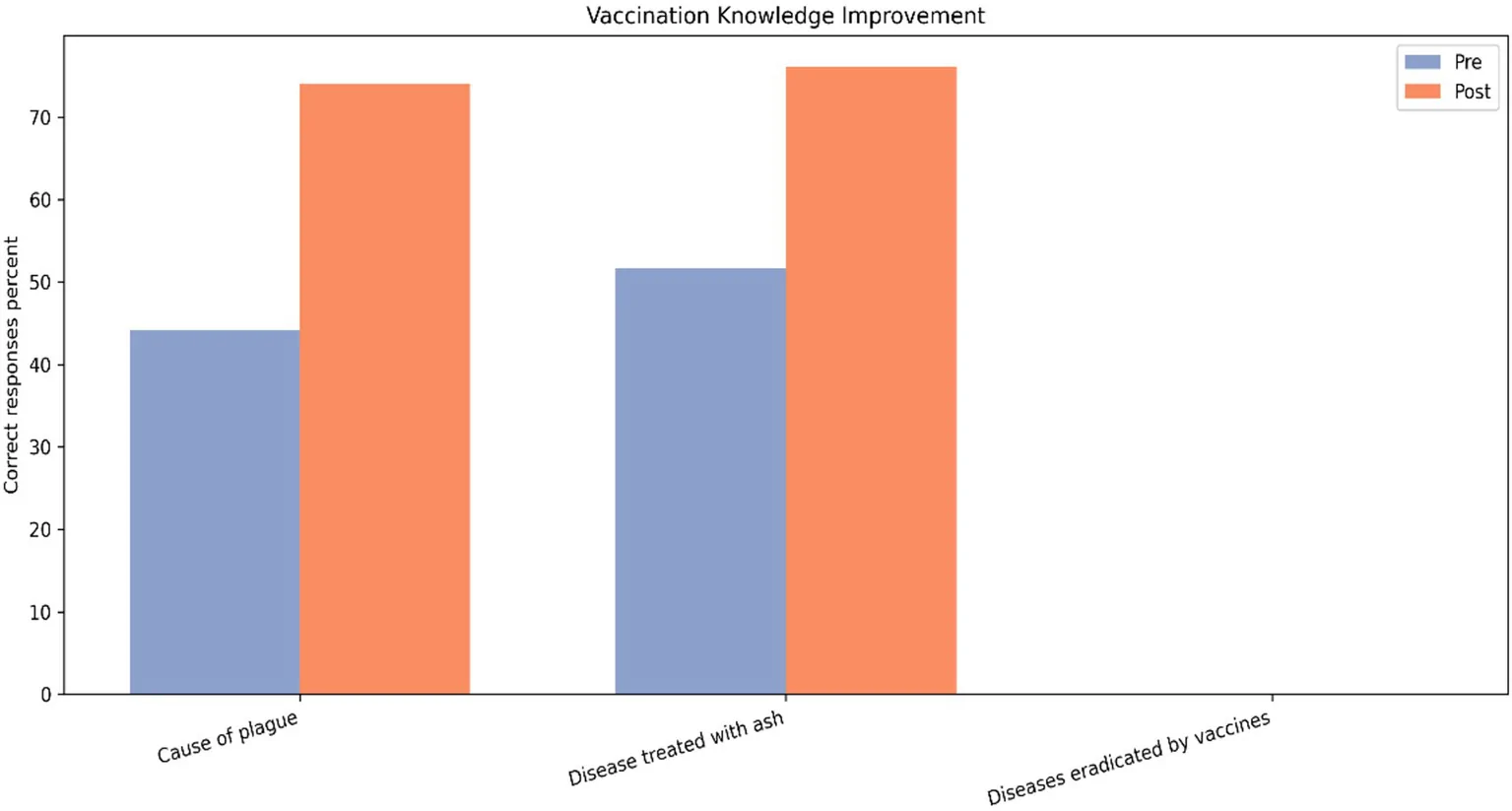
Vaccination knowledge improvement pre and post.

Participants demonstrated significantly improved knowledge following the exhibition. Correct identification of the cause of plague increased from 44.2 to 74.0% (*p* < 0.001), knowledge regarding the disease historically treated with ash increased from 51.7 to 76.1% (*p* < 0.001), and awareness of vaccine-eradicated diseases increased from 39.3 to 64.0% (*p* < 0.001; Table 3; Figure 1).

**Table 3:** Changes in vaccination knowledge before and after the exhibition.

Knowledge item	Correct pre *n* (%)	Correct post *n* (%)	Test	Statistic	*p*-value
Cause of plague	172 (44.2)	288 (74.0)	McNemar	78.72	<0.001
Disease treated with ash	201 (51.7)	296 (76.1)	McNemar	63.57	<0.001
Diseases eradicated by vaccines	153 (39.3)	249 (64.0)	McNemar	58.75	<0.001*

**Figure 2:**
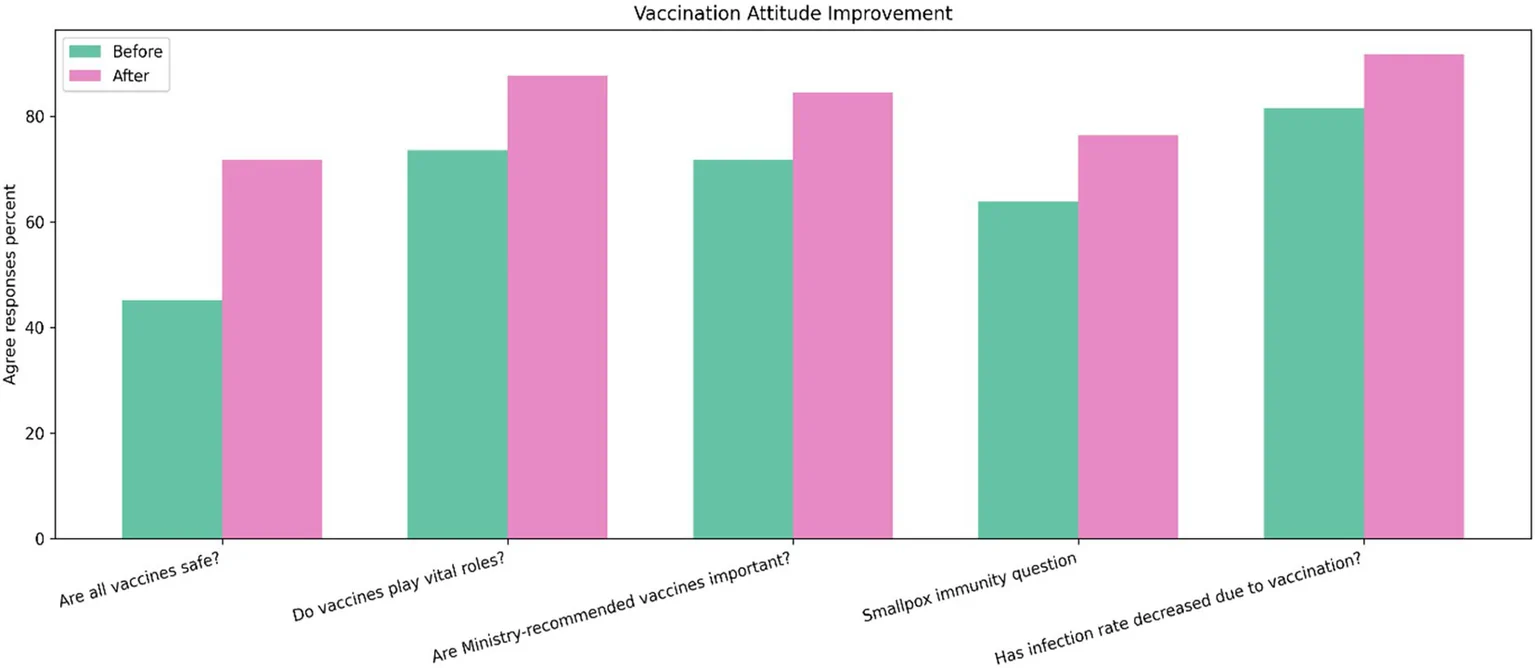
Vaccination attitude improvement pre and post.

Wilcoxon signed-rank testing demonstrated statistically significant improvements in participants’ attitudes toward vaccination across all assessed domains (all *p* < 0.001). Participants reported greater confidence in vaccine safety, stronger agreement regarding the role of vaccines in disease prevention, and increased recognition of the public health benefits of vaccination after attending the exhibition (Tables 4–6; Figure 2).

**Table 4:** Changes in vaccination attitudes before and after the exhibition.

Attitude item	Test	Statistic	*p*-value
Are all vaccines safe?	Wilcoxon signed-rank	1,452.0	<0.001
Do vaccines play vital roles?	Wilcoxon signed-rank	763.5	<0.001
Are ministry-recommended vaccines important?	Wilcoxon signed-rank	1,404.0	<0.001
Smallpox immunity question	Wilcoxon signed-rank	2,731.0	<0.001
Has infection rate decreased due to vaccination?	Wilcoxon signed-rank	313.5	<0.001

**Table 5:** Association between demographic characteristics and post-exhibition knowledge scores.

Variable	*H* Statistic	*p*-value
Sex	0.847	0.357
Age group	1.335	0.855
Education level	11.918	0.103
Information source	39.938	0.562

**Table 6:** Association between age group and post-exhibition attitude scores.

Age group	Mean ± SD	*n*
10–20 years	6.20 ± 1.69	109
21–30 years	6.06 ± 1.58	169
31–40 years	6.02 ± 1.61	60
41–50 years	5.53 ± 0.88	32
51–60 years	5.32 ± 0.75	19

Kruskal–Wallis: *H* = 8.21, *p* = 0.084.

No statistically significant associations were observed between demographic characteristics and post-exhibition knowledge scores (all *p* > 0.05).

Although younger participants tended to demonstrate slightly higher post-exhibition attitude scores, the difference across age groups did not reach statistical significance (*H* = 8.21, *p* = 0.084).

Although Dunn–Bonferroni pairwise comparisons were conducted as a post-hoc analysis, no significant differences were identified between any age-group pairs after adjustment for multiple comparisons (all adjusted *p* > 0.05; Supplementary Table S1).

### 3.1 Nutritional knowledge and attitudes study (*N* = 162)

**Figure 3:**
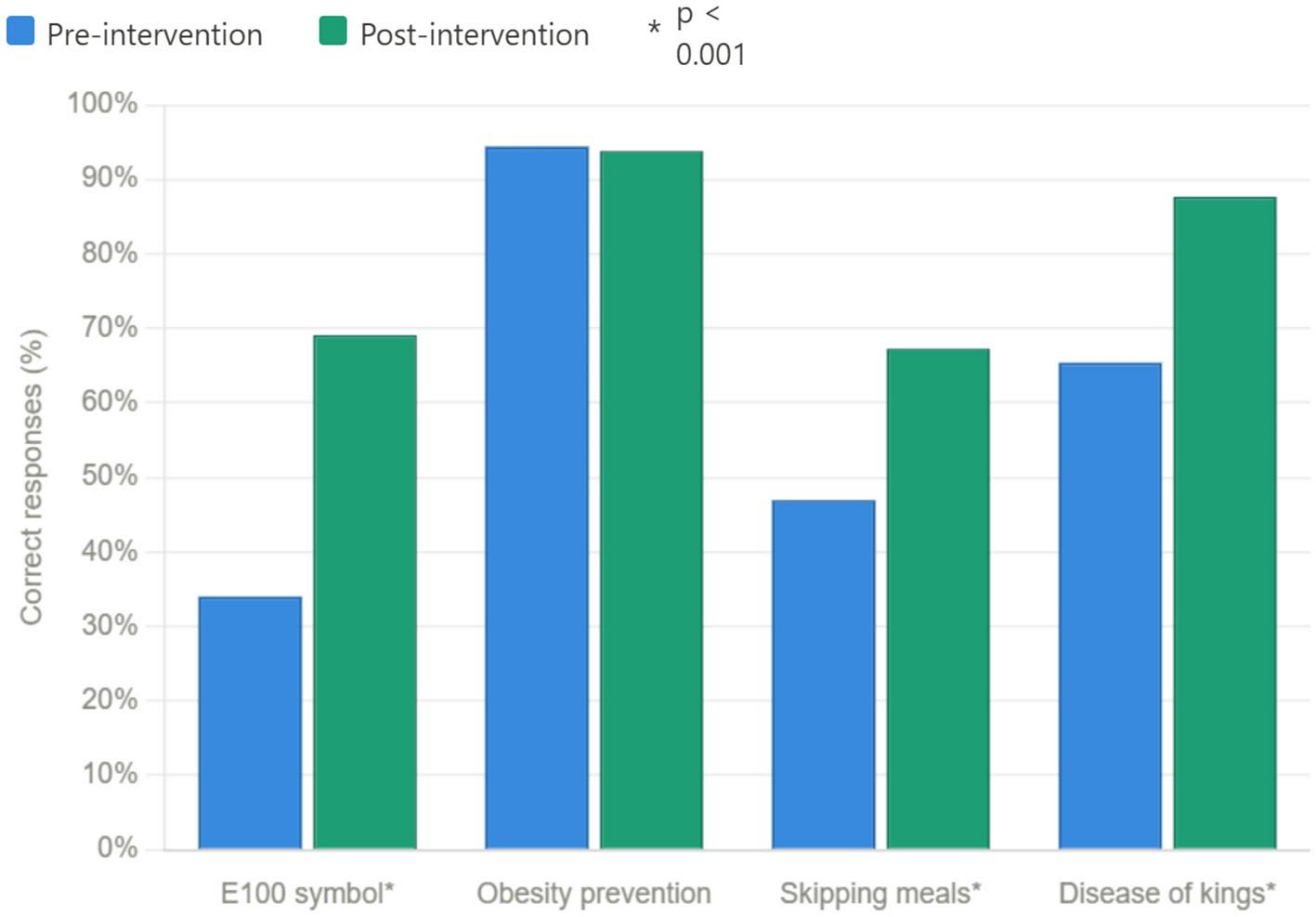
Percentage of correct responses before and after the intervention.

**Figure 4:**
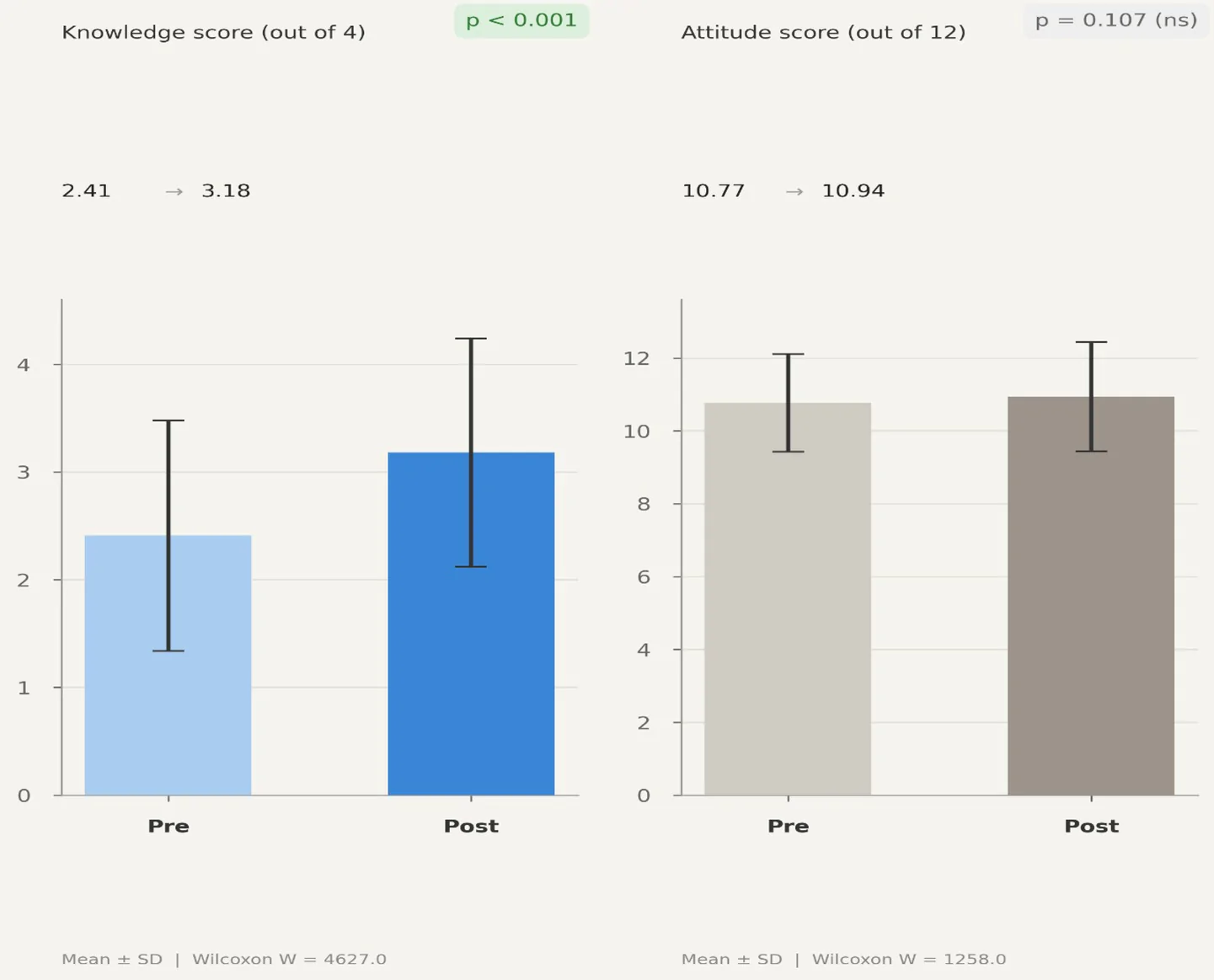
Overall pre and post intervention score presentation.

Figure 3 shows the knowledge improvement after the intervention (Tables 7–12; Figure 4).

**Table 7:** Demographic characteristics (*N* = 162).

Variable	Category	*n*	%
Gender	Male	58	35.8%
	Female	104	64.2%
Age group	10–20	45	27.8%
	21–30	67	41.4%
	31–40	35	21.6%
	41–50	8	4.9%
	More than 50	7	4.3%
Education	Middle school	8	4.9%
	High school	34	21.0%
	Diploma	24	14.8%
	Bachelor’s	81	50.0%
	Master’s	6	3.7%
	PHD	9	5.6%

**Table 8:** Overall knowledge and attitude scores: pre vs. post intervention.

Outcome	Pre mean ± SD	Post mean ± SD	Test	*p*-value
Knowledge score (out of 4)	2.41 ± 1.07	3.18 ± 1.06	Wilcoxon *W* = 4,627.0	<0.001*
Attitude score (out of 12)	10.77 ± 1.34	10.94 ± 1.50	Wilcoxon *W* = 1,258.0	*p* = 0.107

Wilcoxon signed-rank tests were used to compare total pre- and post-intervention scores for both outcomes. Overall knowledge scores improved significantly following the intervention (*W* = 4,627.0, *p* < 0.001). Overall attitude scores showed a small numerical increase but did not change significantly (*W* = 1,258.0, *p* = 0.107). *p < 0.05. Wilcoxon signed-rank test (two-tailed). *N* = 162 for both outcomes.

**Table 9:** Knowledge scores: pre vs post intervention.

Knowledge item	Pre *n* (%)	Post *n* (%)	*χ*^2^ Statistic	*p*-value
E100 = food additive code	55 (34.0%)	112 (69.1%)	45.449	<0.001*
Obesity prevention = portion control + physical activity	153 (94.4%)	152 (93.8%)	0.000	1.000
Skipping meals → low energy and poor sleep	76 (46.9%)	109 (67.3%)	26.256	<0.001*
Gout = ‘disease of kings’	106 (65.4%)	142 (87.7%)	26.630	<0.001*
Total Score (mean ± SD, out of 4)	2.41 ± 1.07	3.18 ± 1.06	–	–

McNemar’s test was used to compare paired proportions of correct responses before and after the intervention. *p < 0.05. The obesity prevention item showed no significant change due to a ceiling effect (pre-intervention accuracy 94.4%).

**Table 10:** Attitude/awareness scores: pre vs post intervention.

Statement	Pre mean ± SD	Post mean ± SD	*W* statistic	*p*-value
Eating small, balanced meals is healthier (positive)	2.69 ± 0.61	2.75 ± 0.57	351.0	0.185
An overweight child is healthy (negative item)	2.54 ± 0.65	2.56 ± 0.72	526.0	0.672
Obesity causes serious health complications (positive)	2.83 ± 0.44	2.88 ± 0.36	117.5	0.196
Protein deficiency can cause health problems (positive)	2.71 ± 0.59	2.75 ± 0.59	178.5	0.354
Total score (mean ± SD, out of 12)	10.77 ± 1.34	10.94 ± 1.50	–	–

*W* = Wilcoxon signed-rank statistic. No item reached statistical significance (all *p* > 0.05). Higher scores reflect more favorable attitudes. The negative item was reverse-scored prior to summation.

**Table 11:** Knowledge score (post) by demographic factors.

Factor	Group	*N*	Mean	SD	Median	Test	*p*
Gender	Male	58	2.93	1.15	3.0	Mann–Whitney *U* = 2,419	0.023*
	Female	104	3.32	0.99	4.0		
Age	10–20	45	2.96	1.22	3.0	Kruskal–Wallis *H* = 4.33	0.228
	21–30	67	3.34	1.02	4.0		
	31–40	35	3.29	0.89	4.0		
	41–50	8	2.88	1.13	3.0		
	More than 50	7	2.86	0.94	3.0		
Education	Middle school	8	3.00	0.76	3.0	Kruskal–Wallis *H* = 9.86	0.043*
	High school	34	3.06	1.18	3.5		
	Diploma	24	2.67	1.31	3.0		
	Bachelor’s	81	3.40	0.92	4.0		
	Master’s	6	3.50	0.84	4.0		
	PhD	9	3.75	0.95	3.5		

The asterisk (*) in Table denotes statistical significance at *p* < 0.05, based on Mann–Whitney *U* test (for gender) and Kruskal–Wallis test (for education level).

**Table 12:** Attitude score (post) by demographic factors.

Factor	Group	*N*	Mean	SD	Median	Test	*p*
Gender	Male	58	10.55	1.81	11.5	Mann–Whitney *U* = 2,516	0.052
	Female	104	11.16	1.26	12.0		
Age	10–20	45	10.67	1.80	11.0	Kruskal–Wallis *H* = 4.30	0.231
	21–30	67	11.21	1.33	12.0		
	31–40	35	10.83	1.48	12.0		
	41–50	8	10.75	1.49	11.0		
	More than 50	7	10.94	1.47	11.0		
Education	Middle school	8	11.38	0.74	11.5	Kruskal–Wallis *H* = 7.43	0.115
	High school	34	10.38	1.89	11.0		
	Diploma	24	10.62	1.50	10.0		
	Bachelor’s	81	11.17	1.37	12.0		
	Master’s	6	11.17	1.17	11.5		
	PhD	9	10.84	1.19	11.0		

### 3.2 Interpretation of demographic comparisons

Post-intervention knowledge scores differed significantly by gender (Mann–Whitney *U* = 2,419, *p* = 0.023), with females scoring higher (median = 4.0) than males (median = 3.0). A significant omnibus difference in knowledge scores was also found across education levels (Kruskal–Wallis *H* = 9.86, *p* = 0.043, valid *n* = 153); however, post-hoc Dunn–Bonferroni correction revealed no individual pairwise comparison remained significant. The smallest adjusted *p*-value was Diploma vs. Bachelor’s (padj = 0.116), suggesting the effect is distributed across groups rather than driven by a single contrast. No significant knowledge differences were found across age groups (*H* = 4.33, *p* = 0.228, valid *n* = 155). Post-intervention attitude scores did not differ significantly by gender (*U* = 2,516, *p* = 0.052), age (*H* = 4.30, *p* = 0.231), or education level (*H* = 7.43, *p* = 0.115). The intervention’s effect on attitudes was broadly consistent across demographic subgroups.

## 4 Discussion

In this study, we examined the short-term effect of health exhibitions on knowledge and attitudes toward nutrition and disease prevention. The findings revealed that both exhibitions contributed to knowledge improvement within the target community, underscoring the role of community health education in improving public awareness. This study addresses the knowledge gap in community interventions by providing a systematic evaluation of how these exhibitions influence public health knowledge and attitudes. Our findings will aid the creation of health plans that address various social and cultural demands. The study also highlights the value of interdisciplinary cooperation in creating a thorough assessment framework that will assist decision-makers and medical professionals in incorporating interactive exhibitions into larger health programs.

This study highlights the impact of information sources on vaccine perception, as seen in the pretest results, where a notable proportion of participants expressed uncertainty about vaccine safety before the exhibition, while many acknowledged the role of vaccines in disease management. The low confidence in vaccine safety was linked to misinformation, with social media being among the most commonly reported sources of vaccination-related information (21.9%, *n* = 85), comparable to healthcare professionals (21.1%, *n* = 82; Table 7). This aligns with previous research, where Loomba et al. (18) found that misinformation reduced vaccine intent. Additionally, Othman et al. (19) and Alaamri et al. (20) confirmed that social media use contributed to vaccine hesitancy in Saudi Arabia. These findings emphasize the need for effective health communication strategies to combat misinformation and enhance vaccine confidence. The observed hesitancy toward vaccines before the exhibition can be explained using the Theory of Planned Behavior (TPB). The initial skepticism among participants, particularly regarding vaccine safety, aligns with the concept of attitudes, where prior exposure to misinformation via social media likely shaped negative perceptions. The subjective norms component also played a role, as social media and peer influence have been documented as key drivers of vaccine hesitancy (18). However, after the exhibition, a significant shift in perception was observed, with increased confidence in vaccines. This can be attributed to an improvement in perceived behavioral control, where participants gained more factual knowledge, reducing uncertainty and empowering them to make informed health decisions.

Vaccination awareness is a crucial factor in controlling the spread of infectious diseases and ensuring public health. A percentage of participants in both the pre- and post-exhibition tests (Table 11) acknowledged the importance of human papilloma virus, shingles, and seasonal flu vaccines, as well as the role of the official vaccination schedule set by the Saudi Ministry of Health, reflecting a strong existing awareness. This suggests that there was already a significant level of agreement before the exhibition, potentially highlighting the impact of the Ministry of Health’s efforts in promoting vaccination through initiatives such as Seasonal Flu Vaccination Campaigns, the Children’s Vaccinations E-Certificate Service, and the Vaccination Follow-up Service (21–23).

Table 10 highlights a significant gap in awareness regarding the causes of the plague among participants, with a large percentage initially lacking this knowledge. However, post-exhibition data indicate a marked increase in awareness, rising to 74%. This improvement is particularly noteworthy in the context of the percentage of participants who accurately identified the disease treated with ash, which surged from 40 to 76.5%. The ability to empower individuals through effective community awareness programs not only influences overall wellbeing but also enables informed health decisions. Such initiatives are essential in bridging knowledge gaps and fostering a more health-conscious society, ultimately contributing to improved health outcomes in the region (24–26).

While vaccine awareness was influenced by external misinformation, a similar pattern was observed in nutrition-related knowledge, where misconceptions about food labeling and meal frequency were prevalent before the exhibition.

There was a significant increase in participants’ awareness of the E100 symbol (from 34 to 69.1%) after the health exhibition, highlighting the effectiveness of educational interventions in improving food labeling-related comprehension. This shift in awareness aligns with the Health Belief Model (HBM), which suggests that individuals are more likely to adopt healthier behaviors when they perceive a risk and recognize the benefits of change. Before the exhibition, misconceptions about the E100 symbol and meal frequency suggested that many participants did not perceive the severity of diet-related health risks. The significant post-exhibition increases in awareness highlights how the exhibition acted as a cue to action, prompting participants to recognize potential health risks and reconsider their dietary choices. Additionally, the increased awareness of obesity-related diseases (from 77.15 to 84.67%) supports the idea that participants began to recognize the benefits of adopting healthier dietary habits. These findings confirm the model’s prediction that providing accessible and engaging health information reduces perceived barriers and increases the likelihood of knowledge uptake and attitude change. McNemar’s test results indicated a statistically significant improvement in participants’ knowledge of the E100 food additive symbol following the exhibition (*p* < 0.001), emphasizing the exhibition’s role in addressing knowledge gaps, especially regarding food labeling literacy. These findings align with Bollinger et al. (27), who found that on-shelf nutritional labeling enhances consumer understanding and influences purchasing decisions. The similarity in outcomes suggests that both exhibitions and product labeling are vital tools for improving food literacy and promoting informed dietary choices, ultimately contributing to better public health.

Misconceptions about meal frequency can impact dietary habits and health. A key finding from the health exhibition was a shift in participants’ views on skipping meals, with most realizing its negative effects on energy and sleep. This aligns with findings reported by Wicherski et al. (28), who found a strong link between skipping breakfast and weight gain due to metabolic disruptions. These results highlight the importance of public health interventions, such as exhibitions and educational programs, in correcting misconceptions and promoting healthier eating habits for better weight management and overall wellbeing.

The findings in (Table 10) reveal a modest increase in participants’ awareness of the benefits of consuming small, balanced meals throughout the day following the exhibition, though this change did not reach statistical significance (*p* = 0.185). This supports existing research on the role of meal timing and frequency in metabolic health. Flanagan et al. (29) emphasize the impact of chrono-nutrition on circadian rhythms and metabolism, while Tinsley and La Bounty (30) highlight how meal frequency influences energy metabolism and body composition, alongside nutrient quality. Additionally, Pradhan et al. (31) provide empirical evidence linking a balanced diet to improved biochemical and physical health markers. These findings reinforce the importance of structured dietary habits in promoting overall health and disease prevention.

After the exhibition, participants showed increased recognition that obesity leads to serious health problems, as reflected by a higher post-exhibition attitude score (Pre mean = 2.83 ± 0.44; Post mean = 2.88 ± 0.36), though this change did not reach statistical significance (*p* = 0.196). The exhibition effectively highlighted the link between obesity and chronic diseases, encouraging participants to understand the long-term effects of unhealthy weight gain.

The findings revealed that a proportion of participants continued to hold the misconception that an overweight child is healthy, with attitude scores showing minimal change (Pre mean = 2.54 ± 0.65; Post mean = 2.56 ± 0.72; *p* = 0.672). These results align with the study by Nobles et al. (32), which emphasizes that public awareness and understanding of childhood obesity’s health risks are still insufficient. While the exhibition successfully raised awareness, a significant portion of participants continued to underestimate the health risks associated with childhood obesity. Nobles et al. (32) argue that childhood obesity is a complex issue influenced by various social, environmental, and behavioral determinants, and that misconceptions about its long-term health consequences can hinder effective preventive strategies. This underlines the need for continued education on the specific health risks related to childhood obesity, as misconceptions may delay early interventions and preventive measures, ultimately leading to serious health complications later in life.

For factors influencing knowledge acquisition in health awareness exhibitions, in both exhibitions, the post-exhibition test evaluations showed that demographic factors did not play a significant role. This can be attributed to several reasons. Firstly, the content of these exhibitions is often designed to be universally accessible, ensuring their relevance across various demographic groups, regardless of age, sex, or educational background. Secondly, the interactive and engaging nature of the exhibitions likely bridged knowledge gaps among attendees, making the experience meaningful for a broad audience. The emphasis on visual and experiential learning may also have contributed to this effect, as such methods tend to be less influenced by demographic differences compared to traditional, text-based approaches such as lectures or written materials. This suggests that the exhibition’s design and delivery methods were key in facilitating knowledge transfer, with less reliance on demographic factors. These findings suggest that interactive exhibitions act as equalizers in knowledge acquisition, making health education more accessible across diverse demographic groups.

Recent research underscores the effectiveness of interactive exhibitions in boosting health awareness, owing to their engaging and immersive qualities. For example, Shwedeh et al. (33) showed that incorporating augmented reality in training programs significantly enhanced knowledge retention and participant involvement, showcasing the benefits of interactive technologies in educational settings. In addition, Chang and Suh (34) demonstrated that integrating virtual reality (VR) into exhibitions enhances visitor engagement and helps them grasp complex health concepts through immersive and interactive experiences. Moreover, Shiner et al. (35) highlighted that healthcare professionals view VR as a valuable tool for both training and patient interaction, underlining its potential to improve health communication and education. Together, these studies affirm that exhibitions, with their interactive, immersive, and emotionally compelling elements, are effective in raising awareness and encouraging improvements in health knowledge and awareness.

A multifaceted, evidence-based strategy that incorporates focused audience research, customized messaging, and successful digital engagement is needed to improve future awareness campaigns. According to recent studies, creating more relevant and resonant health messages can be facilitated by applying data-driven insights to comprehend the cultural and behavioral traits of certain populations (36). Furthermore, using contemporary digital platforms, such as social media, smartphone apps, and online communities, allows for two-way interaction and real-time communication, both of which are essential for fostering trust and improving message retention (37). In addition to raising awareness, adopting an integrated, theory-informed approach may support more targeted health communication strategies aligned with the psychological and cultural factors that influence health decisions. Future studies should consider incorporating follow-up assessments to examine whether short-term knowledge gains translate into sustained awareness over time. Continuity-based strategies—such as digital follow-ups, peer-led community programs, and school-based health integration—may further reinforce the knowledge improvements observed in this study.

This study has some limitations. Firstly, its voluntary nature may lead to self-selection bias; however, efforts were made to encourage diverse participation through targeted outreach to all exhibition attendees. Secondly, the relatively small sample size may limit the generalizability of the findings to the broader population. Lastly, the study was conducted in a single region, which may limit the applicability of the findings to broader or more diverse populations.

## 5 Conclusion

This study demonstrated the positive effect of health exhibitions in enhancing public awareness about vaccination and nutrition in the Qassim region of Saudi Arabia. The results highlight the value of integrating interactive, community-based approaches into national health promotion strategies. Although the findings are rooted in a specific regional context, they offer relevant insights for health education efforts in similar cultural settings. Further research is encouraged to examine long-term knowledge retention and to adapt these methods to broader, more diverse populations.

## Data Availability

The datasets presented in this study can be found in online repositories. The names of the repository/repositories and accession number(s) can be found in the article/Supplementary material.
