# Supplementary material for: Effectiveness of health exhibitions on knowledge and attitudes toward nutrition and disease prevention in Saudi Arabia: an intervention study
Source: Front Public Health. 2026 Aug 7;14:1827711. doi: 10.3389/fpubh.2026.1827711 (PMC13493586; doi:10.3389/fpubh.2026.1827711)
Supplement: Supplementary file 1 [file Data_Sheet_1.docx]

**Questionnaire 1
Enhancing Public Health Awareness: The Effectiveness of Health Exhibitions on Knowledge and Attitudes Toward Nutrition and Disease Prevention**

**Dear Participants,**

We are a research team from the Patients Friends’ Association in Unaizah. We kindly invite you to participate in this pre-and post-survey, which aims to evaluate the effectiveness of health exhibitions in enhancing public health awareness, specifically focusing on the " Health and Nutrition Awareness. "

Your participation is completely voluntary, and all information collected in this survey will remain strictly confidential. The data will only be used for the purposes of this study. The survey will take approximately 2-5 minutes, and no more than 10 minutes to complete. Your help in completing the following survey will be of great scientific and practical value.

For inquiries, you can contact us via email( [info@pf.org.sa](mailto:info@pf.org.sa) )

Thank you

Part one:

1. Gender

- Male
- Female

2. Age

- 10-20 years old
- 21-30 years old
- 31-40 years old
- 41-50 years old
- Over 50 years old

3. Educational level

- Primary school
- Middle school
- High school
- Diploma
- Bachelor’s degree
- Master’s degree
- PhD
- Uneducated

4. What are your primary sources for obtaining information and news?

- Healthcare practitioners (doctor, pharmacist, etc.)
- Social media platforms
- Website
- Family, relatives snd friends
- Other sources

Part two:

5- What are the factors that may contribute to hair and nail loss?

- Genetic
- Vitamin D deficiency
- Protein and iron deficiency
- Vitamin C deficiency

6- The symbol (E100) found on food and medicine packaging stands for what?

- Country of origin
- Product shelf life
- Additives
- I don’t know

7- What is the correct way to prevent obesity?

- Long-term deprivation
- Controlling food portions + physical activity
- Surgical procedures
- I don’t know

8- What are the results of skipping meals, starving the body, and intermittent fasting?

- Achieving a goal without harm
- Low energy and impact on sleep quality
- Increased activity
- I don’t know

9- What is meant by the “disease of kings”?

- Skin discolouration
- Shortness of breath
- Gout
- I don’t know

10- In your opinion, is eating small, balanced meals throughout the day better than having a few heavy meals?

- Agree
- Neutral
- I don’t agree

11. In your opinion, is an overweight child considered to be in good health?

- Agree
- Neutral
- I don’t agree

12. In your opinion, can obesity lead to further complications such as heart disease, diabetes, and breathing problems that affect the quality of life of the affected person?

- Agree
- Neutral
- I don’t agree


  **Questionnaire 2
  Enhancing Public Health Awareness: The Effectiveness of Health Exhibitions on Knowledge and Attitudes Toward Nutrition and Disease Prevention**

**Dear Participants,**

We are a research team from the Patients Friends’ Association in Unaizah. We kindly invite you to participate in this pre-and post-survey, which aims to evaluate the effectiveness of health exhibitions in enhancing public health awareness, specifically focusing on the "Infectious Disease Prevention Awareness."

Your participation is completely voluntary, and all information collected in this survey will remain strictly confidential. The data will only be used for the purposes of this study. The survey will take approximately 2-5 minutes, and no more than 10 minutes to complete. Your help in completing the following survey will be of great scientific and practical value.

For inquiries, you can contact us via email( [info@pf.org.sa](mailto:info@pf.org.sa) )

Thank you.

Part one:

- Gender
- Male
- Female
- Age
- 10-20 years old
- 21-30 years old
- 31-40 years old
- Over 50 years old
- Educational level
- Primary school
- Middle school
- High school
- Diploma
- Bachelor’s degree
- Master’s degree
- PhD
- Uneducated
- What are your primary sources for obtaining information and news?
- Healthcare practitioners (doctor, pharmacist, etc.)
- Social media platforms
- Website
- Family, relatives snd friends
- Other sources

Part two:

- What is the cause of contracting the plague?
- Genetic
- Virus
- Zoonotic bacteria
- Some food
- I don’t know
- It was believed that ash was a cure for which disease?
- Polio
- Measles and smallpox
- Plague
- I don’t know
- What diseases have been eradicated in Saudi Arabia, thanks to God and then vaccines?
- Influenza
- Human papillomvairus (HPV)
- Smallpox and polio
- Shingles
- In your opinion, are all vaccines safe?
- Agree
- Neutral
- I don’t agree
- In your opinion, does the vaccine play a vital role in managing diseases and reducing their complications?
- Agree
- Neutral
- I don’t agree
- In your opinion, are the vaccines recommended by the ministry for HPV, shingles, and seasonal flu important?
- Agree
- Neutral
- I don’t agree

11. In your opinion, were the people who lived during the smallpox epidemic have weak immunity due to the difficulty of the lifestyle at that time?

- Agree
- Neutral
- I don’t agree

12. In your opinion, has the rate of infection with diseases included in the official vaccination schedule by the Saudi Ministry of Health decreased?

- Agree
- Neutral
- I don’t agree
