# Supplementary material for: Effectiveness of health exhibitions on knowledge and attitudes toward nutrition and disease prevention in Saudi Arabia: an intervention study
Source: Front Public Health. 2026 Aug 7;14:1827711. doi: 10.3389/fpubh.2026.1827711 (PMC13493586; doi:10.3389/fpubh.2026.1827711)
Supplement: Supplementary file 2 [file Table_1.docx]

**Supplementary Table S1. Dunn-Bonferroni Pairwise Comparisons of Post-Exhibition Attitude Scores Across Age Groups**

| **Age Group 1** | **Age Group 2** | **Adjusted p-value** |
| --- | --- | --- |
| 10–20 | 21–30 | 1.000 |
| 10–20 | 31–40 | 1.000 |
| 10–20 | 41–50 | 0.635 |
| 10–20 | 51–60 | 0.153 |
| 21–30 | 31–40 | 1.000 |
| 21–30 | 41–50 | 1.000 |
| 21–30 | 51–60 | 0.396 |
| 31–40 | 41–50 | 1.000 |
| 31–40 | 51–60 | 1.000 |
| 41–50 | 51–60 | 1.000 |

Dunn-Bonferroni post-hoc pairwise comparisons following Kruskal-Wallis testing of post-exhibition attitude scores across age groups.

# **Supplementary Table S2. Post-hoc Dunn–Bonferroni Test**

| **Comparison** | **Z** | **p (raw)** | **p (Bonferroni adj.)** | **Sig.** |
| --- | --- | --- | --- | --- |
| Middle school vs High school | 0.691 | 0.489 | 1.000 |  |
| Middle school vs Diploma | 0.131 | 0.896 | 1.000 |  |
| Middle school vs Bachelor's | 1.438 | 0.150 | 1.000 |  |
| Middle school vs Master's | 1.137 | 0.256 | 1.000 |  |
| High school vs Diploma | 1.220 | 0.223 | 1.000 |  |
| High school vs Bachelor's | 1.279 | 0.201 | 1.000 |  |
| High school vs Master's | 0.773 | 0.439 | 1.000 |  |
| Diploma vs Bachelor's | 2.524 | 0.012 | 0.116 |  |
| Diploma vs Master's | 1.463 | 0.144 | 1.000 |  |
| Bachelor's vs Master's | 0.191 | 0.848 | 1.000 |  |

No pairwise comparison reached significance after Bonferroni correction. Diploma vs Bachelor's had the smallest adjusted p-value (p_adj = 0.116).
